# Volume XVI

**Published:** 1877-07

**Authors:** 


### Editorial.

### Volume XVI.

Volumn sixteen is completed by this July number. It is short three numbers, which are deducted in making the price of the volume. Complaint has been made from all quarters, that the Journal has not reached subscribers regularly. We cannot now correct the past, only by deducting the cost of these lost or miscarried numbers from the price of the volumes. It is therefore very desirable that subscribers *reply* to the invitation sent them with their bills; as we now desire to know how our accounts should really stand. It will be the aim of the Journal in future, to be prompt and regular, and to represent the progress of medicine and surgery as truly and fully as possible. Old subscribers and correspondents are cordially invited to contribute to our pages, and not forget us in our efforts to publish the experience and observation of the profession. Index for volume XVI will be found in this number.

Many thanks are due our patrons for their indulgence, and many hopes are entertained for the future of our journal. It is the oldest monthly medical periodical in the state; has a history and hereditary descent to be proud of, and with the favor of the profession, is sure to continue a medium of communication which will be valuable to those interested; but who knows the future. Let us *hope* and *trust.*

